# Acute Kidney Injury Associated with Linagliptin

**DOI:** 10.1155/2016/5695641

**Published:** 2016-02-14

**Authors:** Deepak K. Nandikanti, Elvira O. Gosmanova, Aidar R. Gosmanov

**Affiliations:** ^1^Kidney Specialists of Southern Nevada, Las Vegas, NV 89106, USA; ^2^Nephrology Section, Stratton VA Medical Center, Albany, NY 12208, USA; ^3^Endocrinology Section, Stratton VA Medical Center, Albany, NY 12208, USA

## Abstract

Linagliptin is a dipeptidyl peptidase-IV (DPP-IV) inhibitor that is approved for the treatment of type 2 diabetes mellitus. About 5% of linagliptin is eliminated by the kidneys and no dose adjustment is recommended in kidney impairment. We report a first case of linagliptin-associated acute kidney injury (AKI) in a patient with preexisting chronic kidney disease (CKD). We hypothesize that AKI was due to renal hypoperfusion from linagliptin-induced natriuresis and intravascular volume contraction in the setting of concomitant lisinopril use, which is known to impair autoregulation and potentiate hypotension-induced AKI. It may be prudent to exert caution and closely monitor kidney function when initiating linagliptin in combination with ACE-inhibitors in CKD patients.

## 1. Introduction

Linagliptin is a dipeptidyl peptidase-4 (DPP-4) inhibitor that was approved for the treatment of type 2 diabetes in 2011. A single daily dose of linagliptin is well tolerated [1]. In contrast with other DPP-4 inhibitors, kidney excretion has minimal contribution for linagliptin elimination (~5%) and no dose adjustment is recommended in patients with kidney impairment [2]. Linagliptin has been used in patients with chronic kidney disease (CKD) [3]; however, the safety of linagliptin in combination with renin-angiotensin system (RAS) blockers in CKD patients is unknown. Here, we report a case of linagliptin-associated acute kidney injury (AKI) in a patient with preexisting CKD.

## 2. Case Description

**Figure 1:**
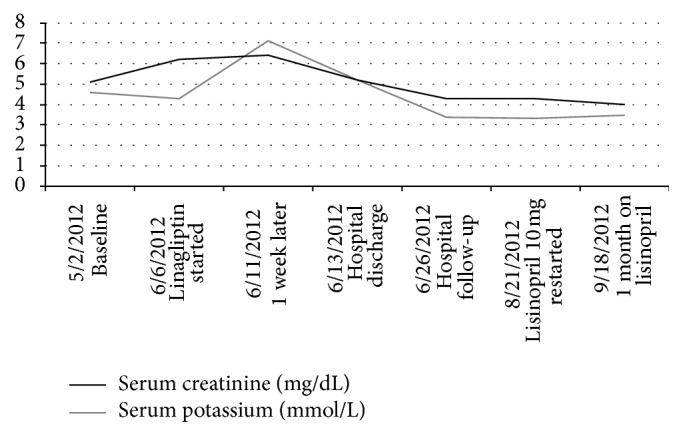
Time-course of changes in serum creatinine and serum potassium in relationship with linagliptin use.

A 54-year-old African-American male with hypertension treated with multiple medications, including lisinopril 80 mg daily, amlodipine 10 mg daily, hydralazine 50 mg trice daily, and clonidine 0.2 mg twice daily; type 2 diabetes controlled with glimepiride 1 mg daily; and stage 4 CKD due to diabetic kidney disease was evaluated in nephrology office during routine follow-up visit. Blood pressure was 156/70 mmHg, which was similar to home measurements. The remaining physical examination was unremarkable. Kidney function was stable with serum creatinine (SCr) of 4.3 mg/dL (estimated glomerular filtration rate (eGFR) of 18 mL/min/1.73 m^2^) and blood urea nitrogen of 64 mg/dL. Potassium level was elevated at 6.4 mmol/L. Hyperkalemia was attributed to several glasses of orange juice that patient was ingesting daily in the last week for the prevention and treatment of recurrent episodes of hypoglycemia. Blood glucose was 70 mg/dL and hemoglobin A1c was 5.5%; therefore, glimepiride was discontinued and linagliptin 5 mg once daily was initiated to reduce the incidence of hypoglycemia. Due to elevated potassium, electrolyte measurement was reassessed one week following linagliptin initiation. At that time, SCr and BUN increased to 7.0 mg/dL and 101 mg/dL, respectively, and hyperkalemia persisted. The patient was admitted to the hospital for evaluation of AKI. On admission, he denied recurrent hypoglycemia, vomiting or diarrhea, or any new medications with the exception of linagliptin. The patient lived with his mother who administered his medications. She denied any changes in compliance and conformed that the patient was in his usual health when he was contacted about elevated SCr. During physical examination blood pressure was 120/57 mmHg with no orthostatic changes. The patient's weight was 2.5 kg lower as compared with his weight in nephrology clinic 1 week ago. Skin turgor was slightly reduced and no signs of peripheral edema were observed. Urinalysis was bland. Fractional excretion of sodium was 3.4%. Linagliptin was discontinued, as the onset of AKI on CKD coincided with linagliptin initiation. Because patient's weight and blood pressure were significantly lower than the usual for him with no signs of infection, the presence of volume depletion was suspected and lisinopril was discontinued. After administration of 2 L of normal saline over 24 hours and oral kayexalate, SCr and potassium levels improved to 5.7 mg/dL and 5.1 mmol/L, respectively. Blood pressure increased to 142/76 mmHg. The patient refused any further interventions and was discharged home. While continuing to hold lisinopril and linagliptin, SCr improved to 3.4 mg/dL in 10 days and remained stable for the next 2 months. Due to chronic proteinuria, a low dose of lisinopril at 10 mg daily was restarted. SCr was unchanged at 3.5 mg/dL 4 weeks following lisinopril initiation (Figure 1). Patient did not require any hypoglycemic medications and was following a diabetic diet.

## 3. Discussion

This report describes patient characteristics and course of AKI after the initiation of linagliptin. The efficacy and safety of linagliptin has been investigated in the recent 52-week study involving diabetic patients with estimated glomerular filtration rate (eGFR) of less than 30 mL/min/1.73 m^2^ [3]. Overall, the incidence of AKI in linagliptin-treated patients was 7.4% and it was similar to the control group. The investigators believed that AKI events were not related to linagliptin; however, no additional details regarding AKI cases were provided. Moreover, it is unknown if linagliptin-treated patients were concomitantly receiving RAS-blockers. In rats, the DDP-4 inhibition downregulates Na^+^/H^+^ exchanger in the proximal renal tubule, which, in turn, leads to natriuresis, diuresis, and attenuation of blood pressure [4, 5]. In the present case, we also observed mild reduction in blood pressure at the time of AKI. While the normal kidney response during hypotension is to preserve sodium, we found an increased fractional urinary excretion of sodium and weight reduction that may be consistent with diuretic effect of linagliptin. In the present case, we hypothesize that AKI occurred from renal hypoperfusion caused by linagliptin-induced natriuresis and intravascular volume contraction in the setting of concomitant lisinopril administration. ACE-inhibitors are known to impair autoregulation and potentiate hypotension-induced AKI [6]. It may be prudent to exert caution when linagliptin is initiated in combination with ACE-inhibitors in patients with limited kidney reserve. Of note, a case of transient AKI associated with linagliptin use was reported in a patient with normal renal function who was also on ACE-inhibitor [7]. Therefore, we recommend to closely monitoring kidney function and blood pressure after linagliptin initiation in diabetic patients with advanced CKD also treated with ACE-inhibitors.
